# Supplementary material for: Bone protection by inhibition of microRNA-182
Source: Nat Commun. 2018 Oct 5;9:4108. doi: 10.1038/s41467-018-06446-0 (PMC6173760; doi:10.1038/s41467-018-06446-0)
Supplement: Supplementary file 1 — Supplementary Information [file 41467_2018_6446_MOESM1_ESM.pdf]

Supplementary Information

Bone protection by inhibition of microRNA-182

Inoue et al.

Supplementary Fig. 1

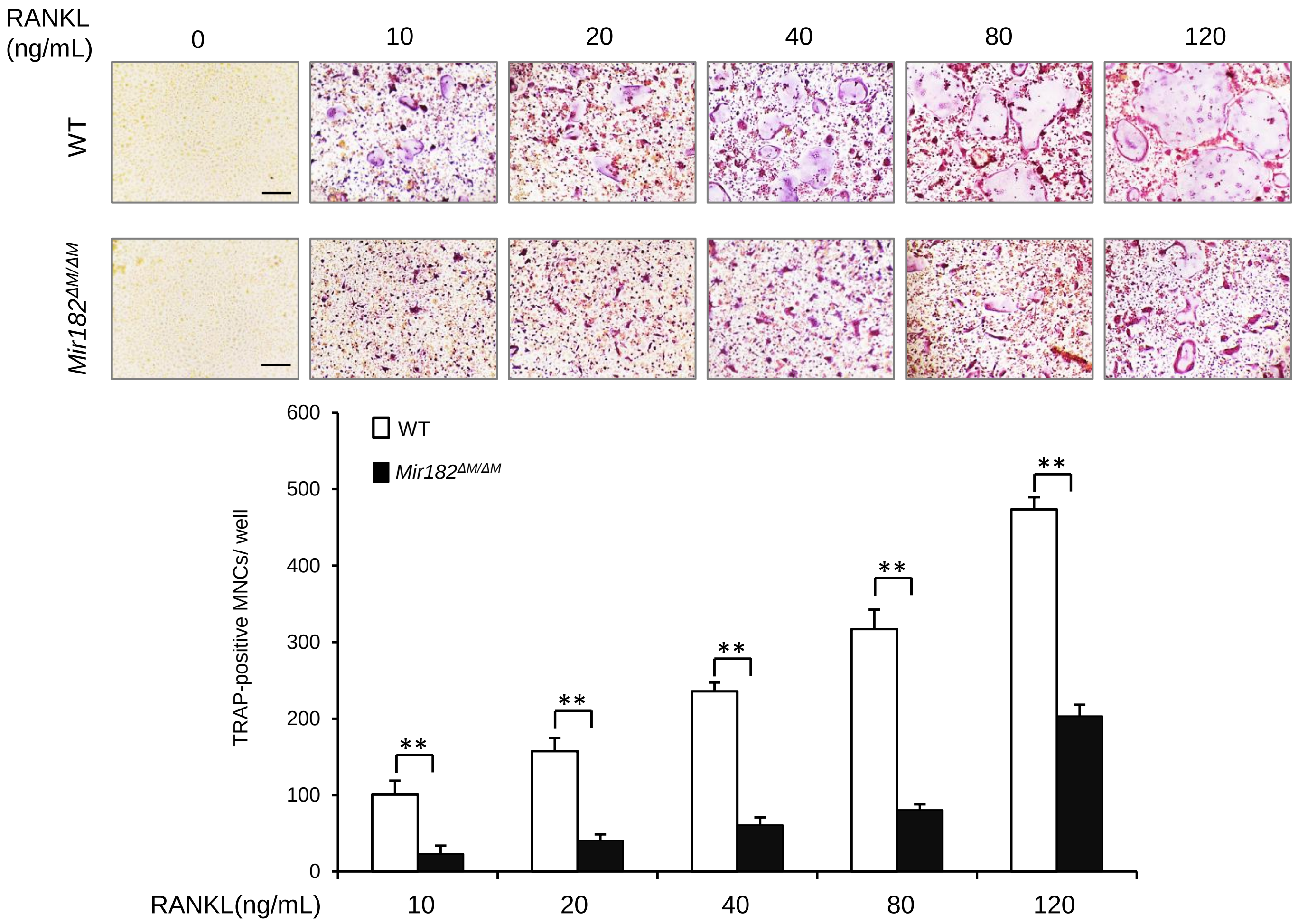

Supplementary Fig. 1 Lack of miR-182 inhibits osteoclastogenesis induced by RANKL at different concentrations. BMMs derived from WT and *Mir182*$^{\Delta M/\Delta M}$ mice were stimulated with different doses of RANKL for three days to induce osteoclast differentiation. TRAP staining (upper panel) was performed and the area of TRAP-positive MNCs (>= 3 nuclei per cell) per well relative to the Control was measured (lower panel). TRAP-positive cells appear red in the photographs. Scale bar, 200 µm. Data are mean ± SD. ***p* < 0.01 by Student's t-test.

Supplementary Fig. 2

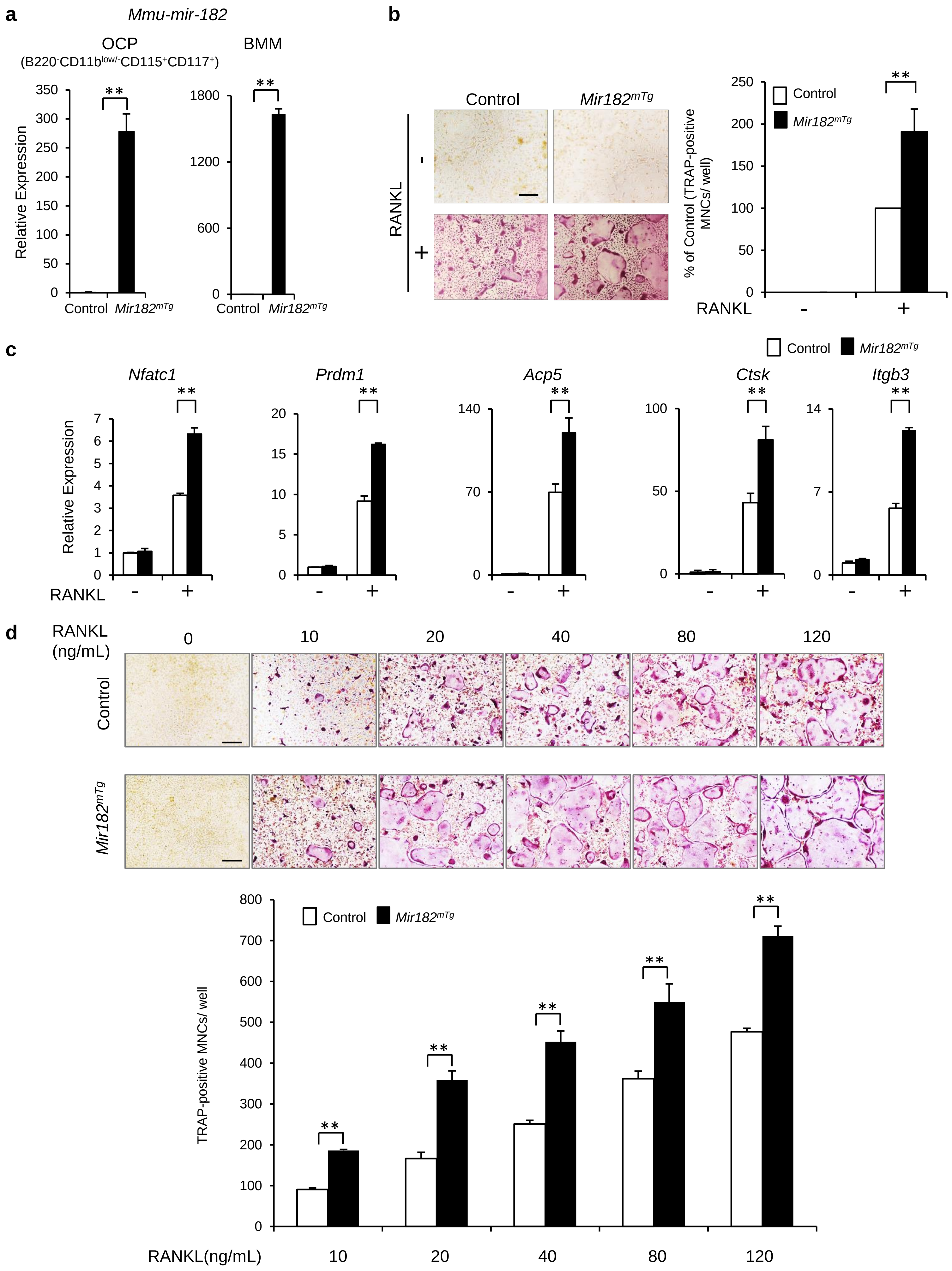

Supplementary Fig. 2 Overexpression of miR-182 promotes osteoclastogenesis *in vitro*. (a) Quantitative real-time PCR analysis of mature mouse miR-182 (*mmu-mir-182*) expression in OCP (B220^{-}CD11b$^{low/-}$CD115(c-Fms)$^{+}$CD117(c-Kit)$^{+}$) isolated from the Control and *Mir182^{mTg}* mice (left panel) and *mmu-mir-182* expression in BMMs derived from the Control and *Mir182^{mTg}* mice (right panel). n=3 per group. (b) Osteoclast differentiation using BMMs derived from the Control and *Mir182^{mTg}* mice stimulated with RANKL for three days. TRAP staining (left panel) was performed and the area of TRAP-positive MNCs (>= 3 nuclei per cell) per well relative to the Control was calculated (right panel). TRAP-positive cells appear red in the photographs. (c) Quantitative real-time PCR analysis of mRNA expression of *Nfatc1*, *Prdm1*, *Acp5, Ctsk*, and *Itgb3* in BMMs from the Control and *Mir182^{mTg}* mice treated with or without RANKL for two days. (d) Osteoclast differentiation using BMMs derived from the Control and *Mir182^{mTg}* mice stimulated with different doses of RANKL for three days. TRAP staining (upper panel) was performed and the area of TRAP-positive MNCs (>= 3 nuclei per cell) per well relative to the Control was measured (lower panel). TRAP-positive cells appear red in the photographs. Scale bars: (b, d) 200 µm. Data are mean ± SD. $^{**}p < 0.01$ by Student's t-test.

Supplementary Fig. 3

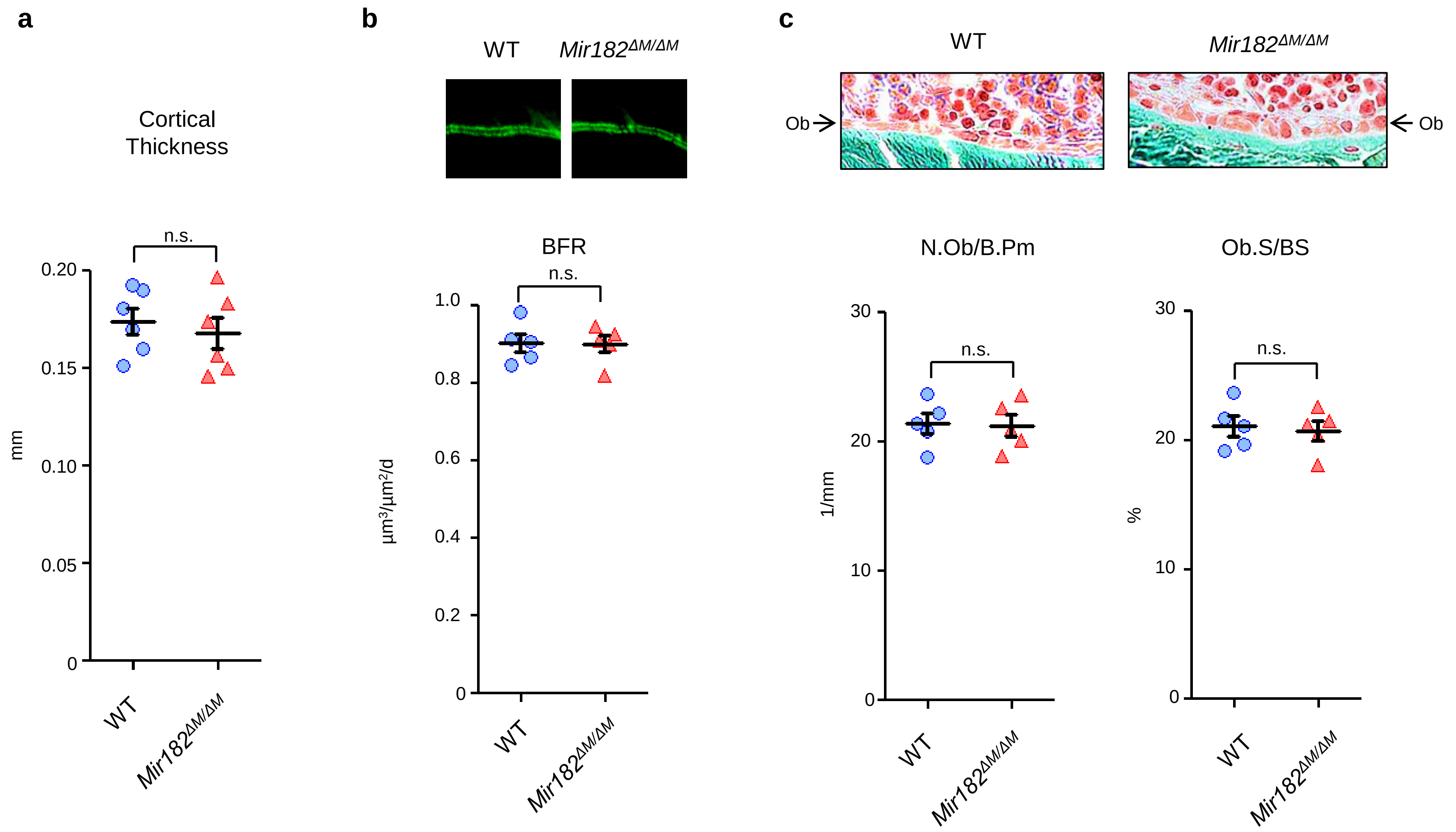

Supplementary Fig. 3 Osteoclastic miR-182 deficiency does not affect bone formation and osteoblastogenesis. (a) Cortical thickness of the midshaft of femurs isolated from 10-week-old male WT and *Mir182*$^{\Delta M/\Delta M}$ littermate mice (n = 6/group). (b) Images of calcein double labeling of tibiae of 10-week-old male WT and *Mir182*$^{\Delta M/\Delta M}$ littermate mice (upper panel, 10x magnification). Histomorphometric quantification of bone formation rate (BFR) in 10-week-old male WT and *Mir182*$^{\Delta M/\Delta M}$ littermate mice (n = 5 per group). (c) Images of Masson-Goldner staining of tibiae of 10-week-old male WT and *Mir182*$^{\Delta M/\Delta M}$ littermate mice (upper panel). The bones show blue, osteoblasts (Ob) on the bone surface appear orange, and bone marrow cells appear red in the photograph. Histomorphometric quantification of N.Ob/B.Pm and Ob.S/BS in 10-week-old male WT and *Mir182*$^{\Delta M/\Delta M}$ littermate mice (n = 5 per group). BFR, bone formation rate; N.Ob/B.Pm, number of osteoblasts per bone perimeter; Ob.S/BS, osteoblast surface per bone surface. (a, b, c) n.s., not statistically significant by Student's t-test, Data are mean ± SEM.

Supplementary Fig. 4

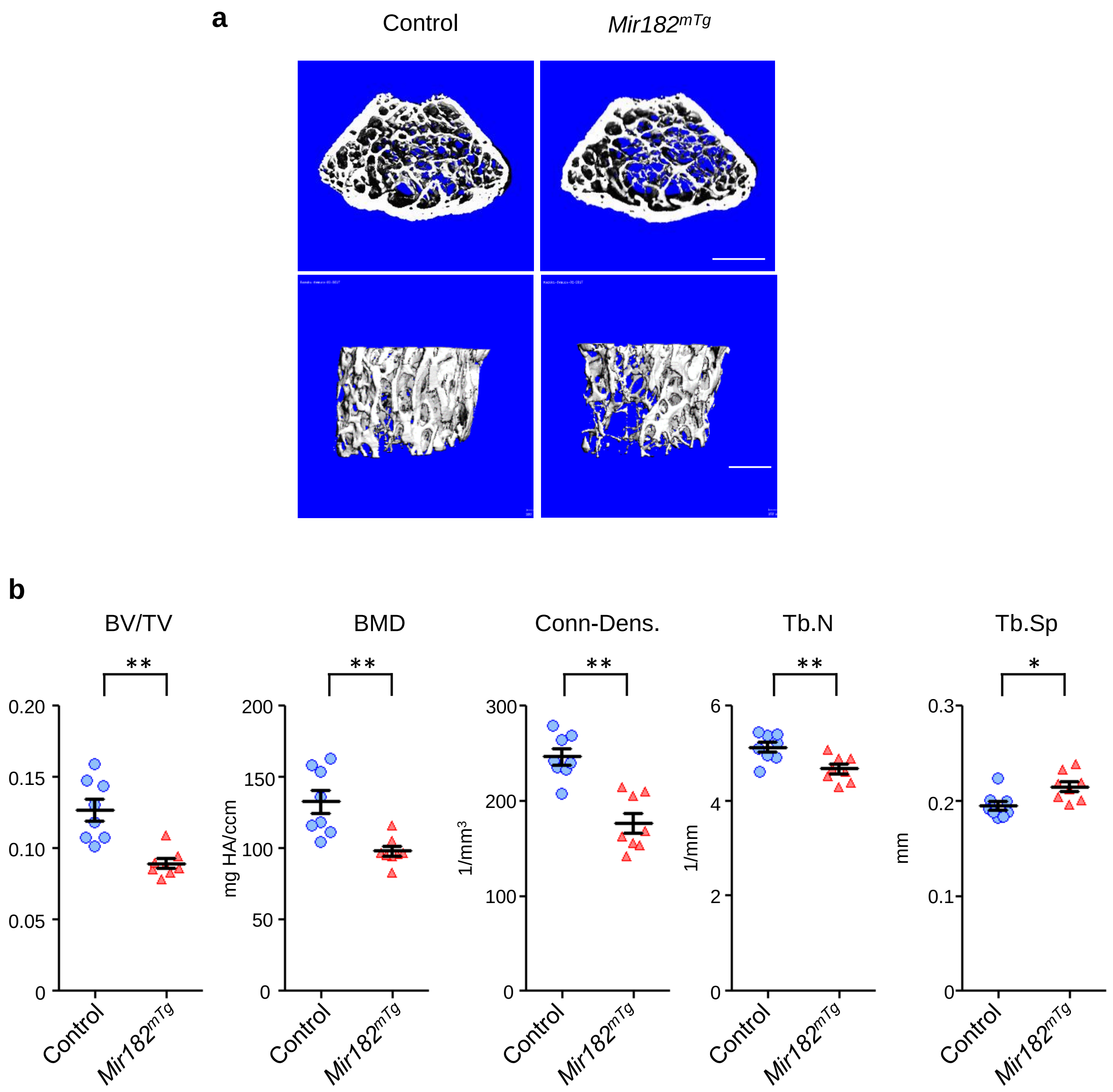

Supplementary Fig. 4 Overexpression of miR-182 leads to osteoporosis in mice. (a) µCT images and (b) bone morphometric analysis of trabecular bone of the distal femurs isolated from 8-week-old male Control and *Mir182*^{mTg} mice (n = 8 per group). Scale bars, 500 µm. *$p < 0.05$; **$p < 0.01$ by Student's t-test. BV/TV, bone volume per tissue volume; BMD, bone mineral density; Conn-Dens., connectivity density; Tb.N, trabecular number; Tb.Sp, trabecular separation.

Supplementary Fig. 5

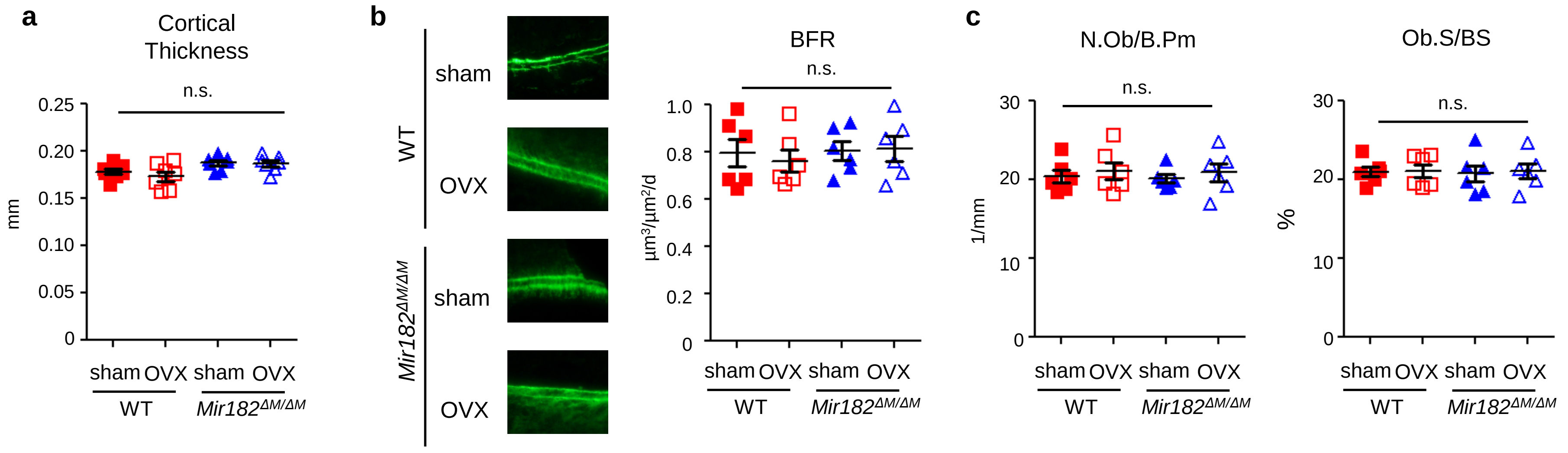

Supplementary Fig. 5 Osteoclastic miR-182 deficiency does not affect bone formation and osteoblastogenesis in OVX model. 10-week-old female WT and *Mir182*$^{\Delta M/\Delta M}$ mice were subjected to OVX or sham surgery and analyzed 5 weeks after surgery. (a) Cortical thickness of the midshaft of femurs isolated from the indicated mice (n = 7/group). (b) Images of calcein double labeling of tibiae of the indicated mice (left panel, 10x magnification). Histomorphometric quantification of BFR in the indicated mice (n = 6 per group). (c) Histomorphometric quantification of N.Ob/B.Pm and Ob.S/BS in the indicated mice (n = 6 per group). (a-c) Data are mean ± SEM. n.s., not statistically significant by two-way ANOVA. BFR, bone formation rate; N.Ob/B.Pm, number of osteoblasts per bone perimeter; Ob.S/BS, osteoblast surface per bone surface.

Supplementary Fig. 6

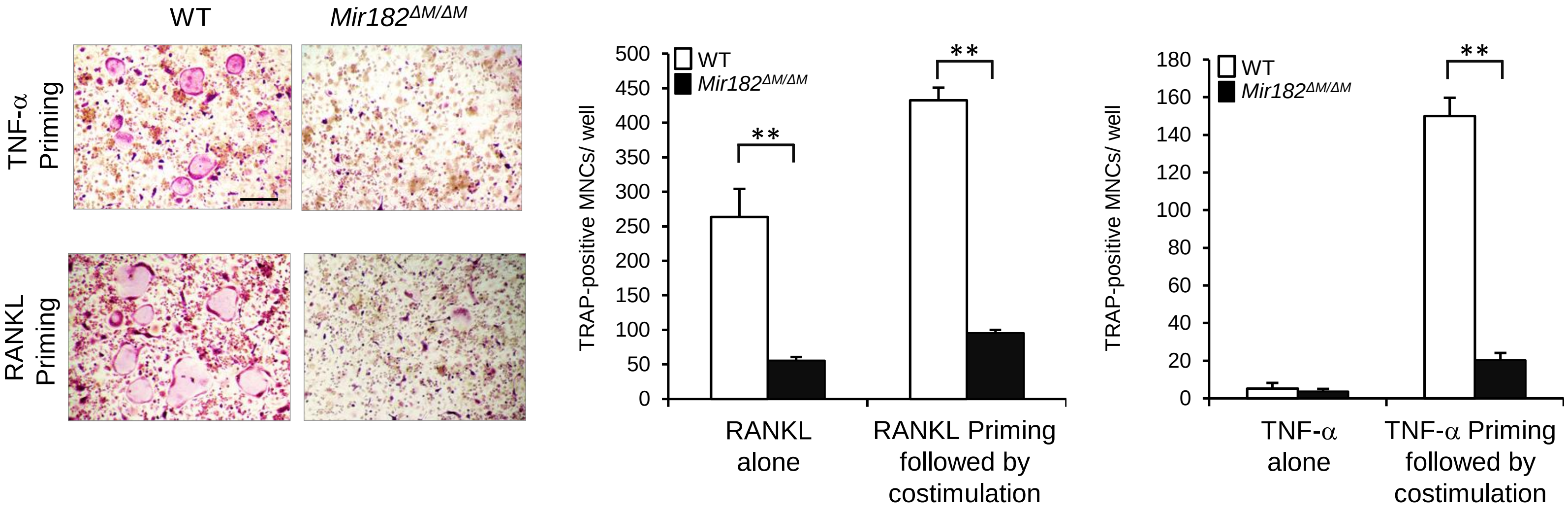

Supplementary Fig. 6 miR-182 deficiency represses osteoclast formation in the TNF-α or RANKL priming conditions. Osteoclast differentiation determined by TRAP staining (left panel) and the TRAP-positive MNCs per well (right panel) in the cell cultures using the WT and *Mir182*$^{\Delta M/\Delta M}$ BMMs that were treated with TNF-α alone (40 ng/ml), RANKL alone or primed with TNF-α (40 ng/ml) or RANKL for 1 day, followed by costimulation with TNF-α and RANKL for 2 days. Data are mean ± SD. $^{**}p < 0.01$ by Student's t-test.

Supplementary Fig. 7

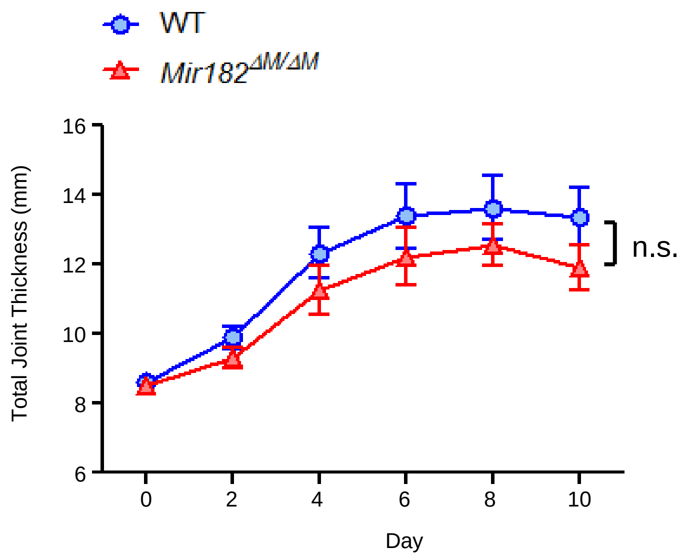

Supplementary Fig. 7 Time course of joint swelling of inflammatory arthritis developed in *Mir182*$^{\Delta M/\Delta M}$ mice and littermate controls. For each mouse, joint swelling was calculated as the sum of measurements of joint thickness of two wrists and two ankles. n = 5 per group. Joint swelling is represented as the mean ± SD for each group. n.s., not statistically significant by Student's t-test.

Supplementary Fig. 8

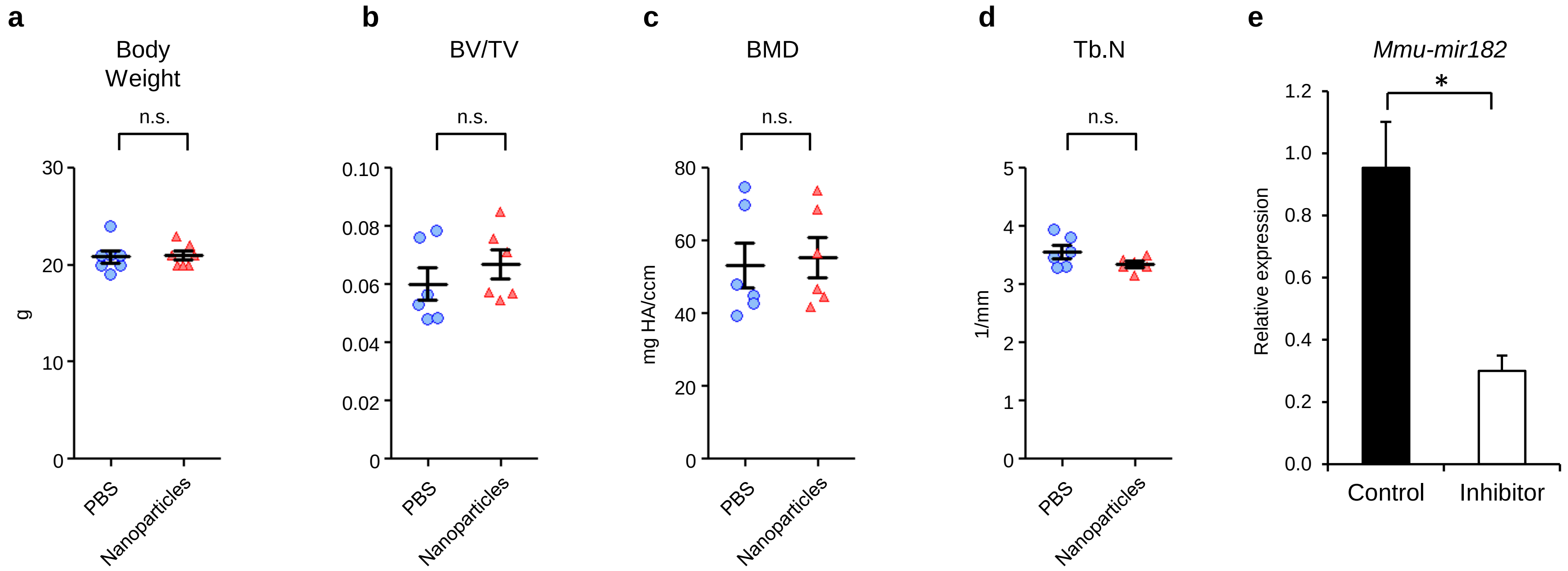

Supplementary Fig. 8 Chitosan nanoparticles per se do not affect either mouse body weight or bone mass. (a) Body weight and (b-d) bone morphometric analysis of trabecular bone of the distal femurs isolated from 15-week-old female C57BL/6 mice treated with PBS or chitosan nanoparticles. PBS (100 µl) or CH nanoparticles (5 µg/100 µl) were delivered by intravenous injections twice a week for 5 weeks. BV/TV, bone volume per tissue volume; BMD, bone mineral density; Tb.N, trabecular number. n = 6 per group. n.s., not statistically significant. (e) Quantitative real-time PCR analysis of mature mouse miR-182 (*mmu-mir-182*) expression in bone marrow isolated from the femurs of mice treated with CH nanoparticles containing the control or miR-182 inhibitor (5 ug per mouse) twice a week for 5 weeks. *$p < 0.05$ by student's t-test. Data are mean ± SD.

Supplementary Fig. 9

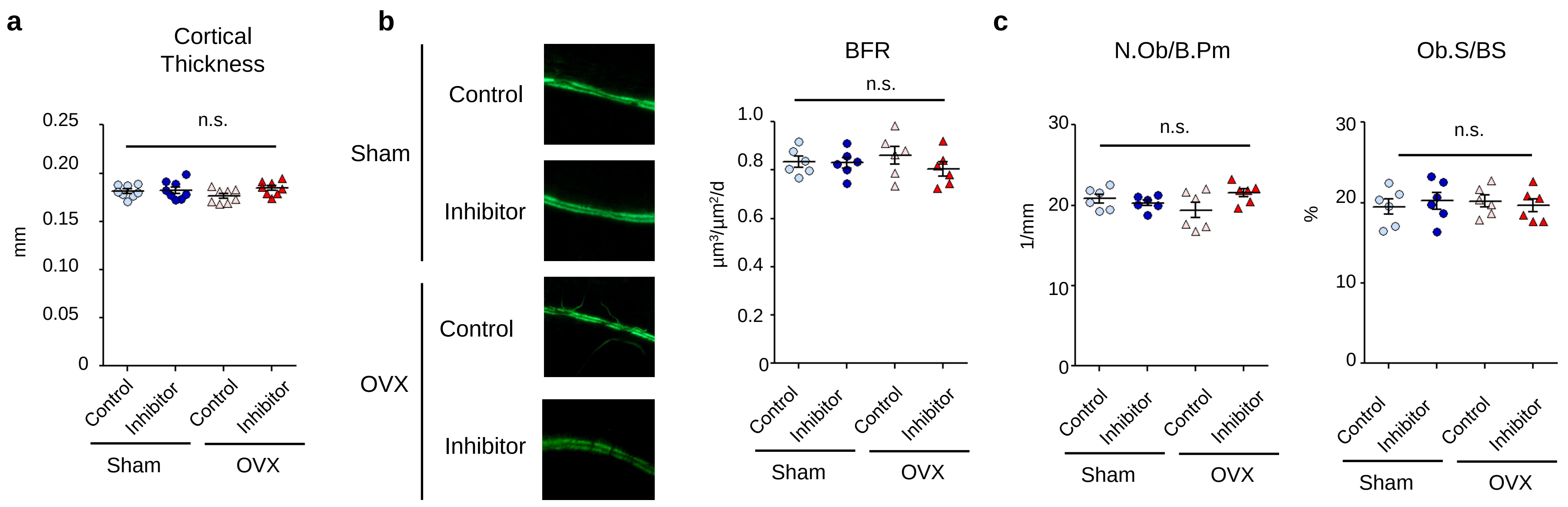

Supplementary Fig. 9 Pharmacological inhibition of miR-182 does not significantly affect bone formation and osteoblastogenesis in OVX model. OVX or sham operation was performed on 10-week-old female C57BL/6 mice. Three days after surgery, the OVX and sham mice were treated with Control or miR-182 inhibitor-carrying CH nanoparticles at 5 μg per mouse twice a week for 5 weeks. (a) Cortical thickness of the midshaft of femurs isolated from the indicated mice (n = 8 per group). (b) Images of calcein double labeling of tibiae of the indicated mice (left panel, 10x magnification). Histomorphometric quantification of BFR in the indicated mice (n = 6 per group). (c) Histomorphometric quantification of N.Ob/B.Pm and Ob.S/BS in the indicated mice (n = 6 per group). (a-c) Data are mean ± SEM. n.s., not statistically significant by two-way ANOVA. BFR, bone formation rate; N.Ob/B.Pm, number of osteoblasts per bone perimeter; Ob.S/BS, osteoblast surface per bone surface.

Supplementary Fig. 10

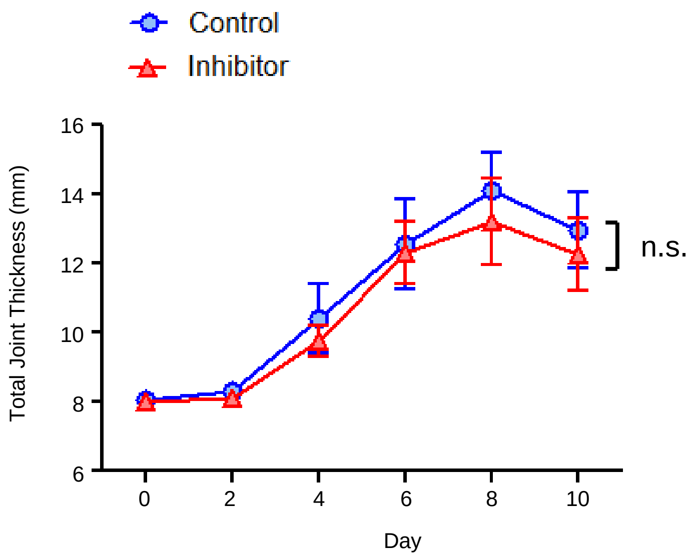

Supplementary Fig. 10 Time course of joint swelling of serum-induced arthritis in C57BL/6 mice treated with the indicated CH nanoparticles. Control or miR-182 inhibitor-carrying CH nanoparticles were delivered by intravenous injections at 5 μg per mouse on day 2, 4, 6, 8. For each mouse, joint swelling was calculated as the sum of measurements of joint thickness of two wrists and two ankles. n = 10 per group. Joint swelling is represented as the mean ± SD for each group. n.s., not statistically significant by Student’s t-test.

Supplementary Fig. 11

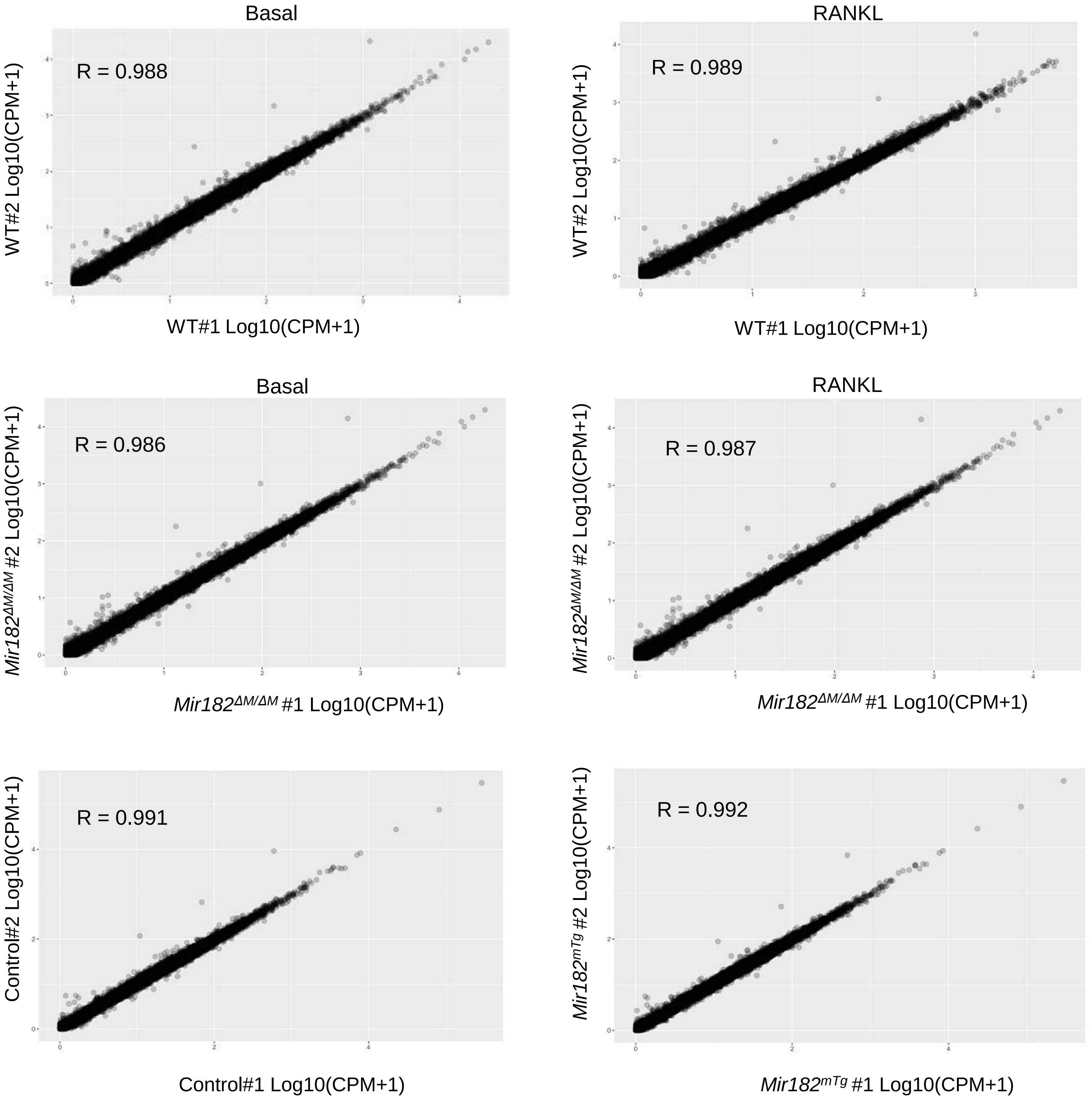

Supplementary Fig. 11 Assessment of reproducibility between two RNA-seq biological replicates. Correlation Plots of log10(CPM+1) for each replicate for Basal-WT (WT cells at basal level), RANKL-WT (WT cells with RANKL stimulation), Basal- *Mir182*$^{\Delta M/\Delta M}$ (*Mir182*$^{\Delta M/\Delta M}$ cells at basal level), RANKL- *Mir182*$^{\Delta M/\Delta M}$ (*Mir182*$^{\Delta M/\Delta M}$ cells with RANKL stimulation), Basal-Control (WT control cells at basal level) and Basal- *Mir182*^{mTg} (*Mir182*^{mTg} cells at basal level) are shown, respectively. Values of Pearson’s correlation coefficient (R) were calculated and plots were generated using the R statistical package. CPM, counts per million mapped reads. #1, replicate 1. #2, replicate 2.

Supplementary Fig. 12

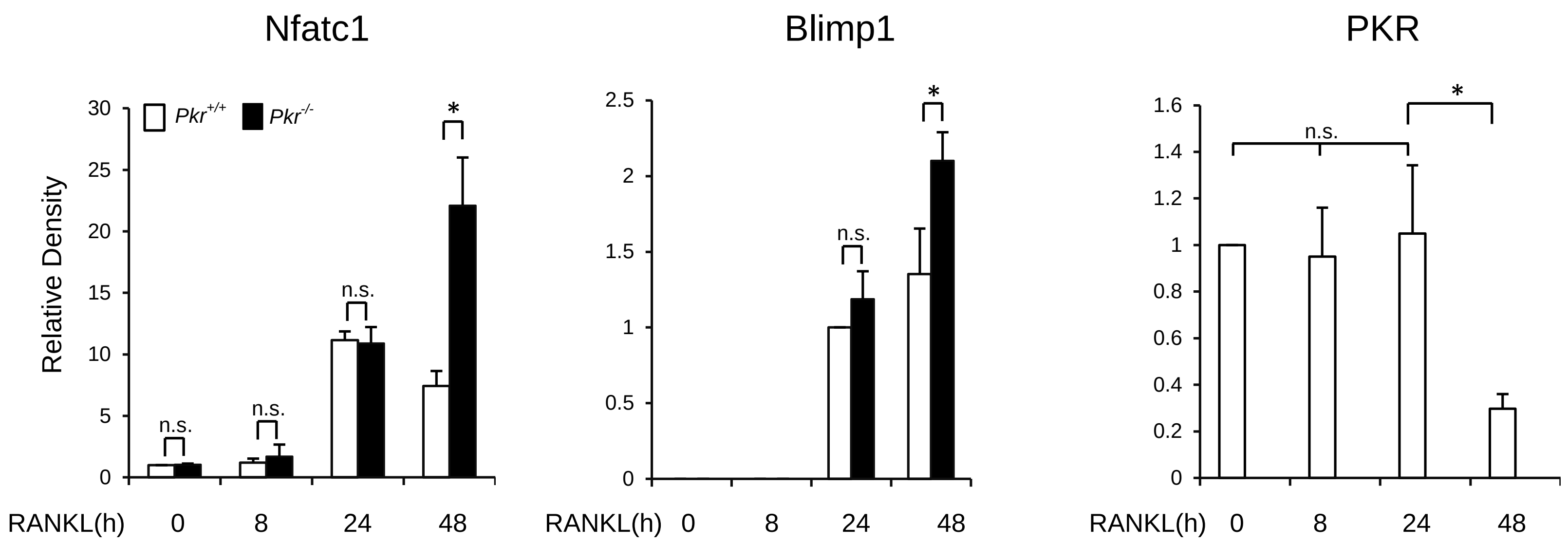

Supplementary Fig. 12 The relative density of immunoblot bands. The relative density of immunoblot bands of NFATc1, Blimp1 and PKR versus those of loading control p38 from three independent experiments were quantified by densitometry as indicated in Methods. *$p < 0.05$; **$p < 0.01$; n.s., not statistically significant by Student’s t-test.

Supplementary Fig. 13

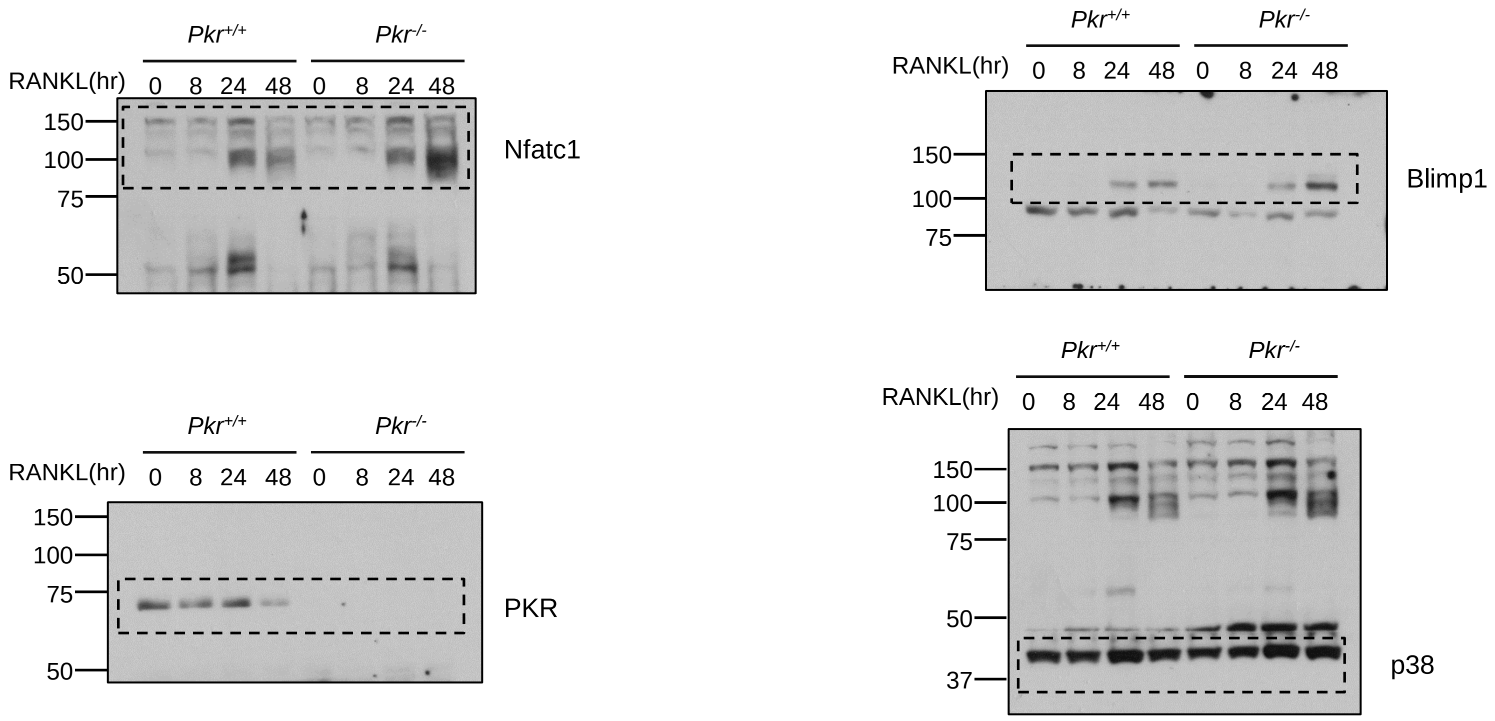

Supplementary Fig. 13 Uncropped scans of Western blots.

Supplementary Methods

Osteoclast Precursor (OCP) Sorting

Mouse bone marrows were isolated from tibiae and femora of the Control and *Mir182*^{mTg} using FACS buffer (PBS (pH 7.2) containing 2% FBS and 2 mM EDTA). The cell suspensions were washed with FACS buffer and filtered with 70 μm cell strainer twice and 40 μm cell strainer once. After washing, equal numbers of cells per mouse were blocked with Purified Rat Anti-Mouse CD16/CD32 (BD Biosciences) for 15 minutes on ice, then stained with BUV395-conjugated Rat Anti-Mouse CD45R/B220 antibody (BD Horizon 562537, Clone RA3-6B2, 1:200), PerCP-Cy™5.5-conjugated Rat Anti-CD11b antibody (BD Pharmingen 550993, Clone M1/70, 1:200), APC-conjugated CD115 (c-fms) antibody (Thermo Fisher Scientific 17-1152-82 , AFS98, 1:200) and PE-conjugated Rat Anti-Mouse CD117(c-kit) antibody (BD Pharmingen 553355, Clone 2B8, 1:200) for 45 minutes on ice. After washing cells twice with FACS buffer, cells were resuspended with FACS buffer and 1μg/mL 4-6-Diamidino-2-Phenylindole (DAPI) (live/dead exclusion) for cell sorting. Cell sorting was performed with a FACS Aria II SORP cell sorter (Becton Dickinson) at Weill Cornell Medical College, with exclusion of DAPI^{+} cells and doublets. B220^{-} CD11b$^{low/-}$ CD115(c-Fms)$^{+}$CD117(c-Kit)$^{+}$ cells were sorted as OCP[1, 2, 3, 4] and subjected to miRNA isolation.

Supplementary References

1. Arai, F., *et al.* Commitment and differentiation of osteoclast precursor cells by the sequential expression of c-Fms and receptor activator of nuclear factor kappaB (RANK) receptors. *The Journal of experimental medicine* **190**, 1741-1754 (1999).
2. Charles, J.F., *et al.* Inflammatory arthritis increases mouse osteoclast precursors with myeloid suppressor function. *The Journal of clinical investigation* **122**, 4592-4605 (2012).
3. Jacome-Galarza, C.E., Lee, S.K., Lorenzo, J.A. & Aguila, H.L. Identification, characterization, and isolation of a common progenitor for osteoclasts, macrophages, and dendritic cells from murine bone marrow and periphery. *Journal of bone and mineral research : the official journal of the American Society for Bone and Mineral Research* **28**, 1203-1213 (2013).
4. Xiao, Y., *et al.* Osteoclast precursors in murine bone marrow express CD27 and are impeded in osteoclast development by CD70 on activated immune cells. *Proceedings of the National Academy of Sciences of the United States of America* **110**, 12385-12390 (2013).
